# Virtual Academic Asynchronous Mentoring (VAAM) for Faculty Physicians: An Innovative Mentorship

**DOI:** 10.7759/cureus.51289

**Published:** 2023-12-29

**Authors:** Mingyuan Yin, Mindy McManus, Nancy Dawson, Leila Tolaymat, Cara C Prier, Winston Tan, Ingrid Pritchard, Ebone Hill, Claire Haga, Mary S Hedges

**Affiliations:** 1 Research Administration, Mayo Clinic, Jacksonville, USA; 2 Human Resources, Mayo Clinic, Jacksonville, USA; 3 Medicine, Mayo Clinic, Jacksonville, USA; 4 Dermatology, Mayo Clinic, Jacksonville, USA; 5 Family Medicine, Mayo Clinic, Jacksonville, USA

**Keywords:** faculty development, mentorship, professional development, academic advancement, virtual mentoring

## Abstract

Introduction: Successful mentorship programs in academic medicine correlate with increased achievement in scholarly activities, leadership, and academic advancement for faculty members, as well as reduced burnout. Despite these benefits, the traditional mentorship model may be underutilized due to challenges of time constraints and alignment in goals. Furthermore, women and underrepresented in medicine (UriM) physicians are less likely to have mentorship, perpetuating the gap in the diversity of academic faculty in leadership and career advancement. To address this, we created an innovative mentorship model for busy academic faculty physicians using a virtual academic asynchronous mentoring video platform.

Methods: A series of videos were created by interviewing 10 identified mentors (four male, six female) from various medical specialties at a national academic institution. The mentors included nine physician faculty with the academic rank of Associate Professor or full Professor and one Research Administrator. Key learning points shared by mentors included topics on academic advancement, mentorship development, leadership development, and research resources.

Results: Between March 2020 and September 2023, the Virtual Academic Asynchronous Mentoring (VAAM) Video Series garnered 182 unique viewers, received 2,107 visits, and accumulated 1,871 total minutes of viewing time. All viewers were surveyed, with an 11% survey response rate received. Fifty-two percent of survey respondents reported that the video content was excellent and 43% reported very good. Seventy-six percent of respondents thought the video series had the potential to enhance their professional development and academic productivity.

Conclusion: The VAAM Video Platform offers a novel approach to academic mentoring for faculty physicians which eliminates limitations of traditional mentorship models in a convenient and cost-effective way. VAAM offers an egalitarian starting point for all junior faculty who have not yet established a mentoring relationship to seek information and resources on academic advancement and career development.

## Introduction

Faculty members in academic medicine often face a unique set of challenges in juggling multiple roles in patient care, teaching, research, and administrative responsibilities, navigating the complex and evolving healthcare system and academic environments, and fulfilling expectations for academic advancement and promotion [1,2]. Effectively addressing these challenges often requires institutional support, mentorship, and ongoing professional development opportunities.

Many studies have shown that successful mentorship programs not only correlate with increased achievement in scholarly activities, leadership, and academic advancement for faculty members, but also mitigate burnout and enhance relatedness, which contribute to higher levels of faculty satisfaction, increased productivity, and reduced turnover rate [3-7]. Despite these benefits, women are less likely to have academic mentorship relationships in the traditional model compared to men. This may relate to the recognized “gender gap” among leadership in academic medicine [8,9]. Based on the 2018-2019 Association of American Medical Colleges (AAMC) report, men still dominate faculty leadership positions in healthcare [10]. Lacking women role models in senior leadership positions could limit the mentoring pool for junior women faculty, as many women favor mentors of the same gender who understand and support their unique paths and challenges as physicians while balancing other responsibilities [9,11].

Similar challenges are faced by physicians who are underrepresented in medicine (URiM). Despite continued increases in ethnic diversity in the US population, Hispanic, Native Hawaiian/or Other Pacific Islander, American Indian/or Alaska Native, and Black physicians continue to have disproportionately lower representation in the US physician workforce compared to white physicians (6.9%, 0.1%, 0.3%, 5.7% versus 63.9 % respectively) [12]. This discrepancy is even more prominent in academic medicine, including medical teaching and research [13]. Based on data from the AAMC, the proportion of full-time URiM women in academic medicine only increased from 12% in 2009 to 13% in 2018 [10]. The greatest proportion of these women faculty were at the rank of assistant professor [10]. Studies reviewing minority faculty development programs have shown that mentorship and faculty development for minority faculty could increase retention, academic productivity, and promotion rates for this group [14]. A study reviewing a range of successful mentorship programs involving URiM physicians showed that a lack of racial concordance between mentor and mentee did not adversely impact satisfaction with or success of these mentorship programs [15].

Traditional mentorship in healthcare settings typically involves an experienced mentor guiding and sharing knowledge with a mentee to facilitate skill development and professional growth [16]. Various mentoring styles, such as one-on-one meetings, shadowing, and networking, are commonly employed within workplaces to foster learning and development [16]. Mentorship is recognized as a bidirectional process, bringing growth opportunities and satisfaction that benefit both parties. Nevertheless, the traditional mentorship approach is not without its limitations. Senior faculty mentors are often busy and face challenges in scheduling time for mentoring responsibilities. In addition, it may be difficult to identify mentors with aligned goals and expertise to fulfill the desired mentorship goals. Consequently, the traditional mentorship model may be underutilized. Thus, there has been interest in developing more flexible and innovative mentoring approaches.

With the advancement of modern technology, virtual mentoring has become a promising alternative to traditional in-person mentorship [17,18]. Several healthcare organizations have sought to arrange various formats of virtual mentorship programs. In 2017, a successful program for resident mentorship utilized a multi-pronged approach including an online toolkit with eLearning modules [19]. In 2018, the American Nurses Association (ANA) launched a national virtual mentorship program in response to a needs assessment for positive and effective mentoring, with survey data showing that the overwhelming majority reported satisfaction with the program and also noted that mentors have a strong “desire to give back” [20]. A study in 2020 described an online international mentoring program focusing on teaching and research skills and noted that it could provide support that otherwise may not be available to new faculty at geographically distant institutions [21]. The COVID-19 pandemic further accelerated the adoption of virtual formats in various aspects of medical education, patient care, faculty development, and mentorship [22-26]. In educational and healthcare settings where real-time communication can be challenging, asynchronous modalities can be employed to foster meaningful mentorship experiences [27].

To explore the potential value of academic mentoring in virtual platforms for faculty physicians at an academic institution, we developed a Virtual Academic Asynchronous Mentoring (VAAM) video series consisting of video interviews conducted with experienced and academically accomplished faculty. Herein, we describe this innovative approach of virtual mentoring to provide effective academic mentoring.

## Materials and methods

Mentor selection process

To address the need for a novel virtual tool to facilitate virtual mentoring, a planning committee was formed comprising the Associate Chair for Faculty Development and a Senior Advisor from Human Resources (HR), both with expertise in physician mentoring. The planning group identified and recruited 10 mentors, consisting of nine physician faculty with the academic rank of Associate Professor or full Professor and one Research Administrator. The 10 mentors (four male and six female) represented various medical specialties across Mayo Clinic and were from the Jacksonville, Florida, and Rochester, Minnesota campuses. The medical specialties represented were Hematology/Oncology, Psychiatry, Pathology, Cardiology, Hospital Internal Medicine, Dermatology, Neurosurgery, and Research Administration.

Topics identified

The planning committee generated a comprehensive list of commonly asked questions during previous mentorship meetings. The planning committee requested feedback and comments on the proposed questions from the 10 mentors participating in the program and made necessary adjustments to the questions based on their input. The planning committee met individually with each of the mentors to review the finalized questions prior to video filming. Key learning points shared by mentors included topics on academic advancement, mentorship development, leadership development, and research resources (Table 1).

**Table 1:** Key Learning Points shared by mentors in VAAM VAAM: Virtual Academic Asynchronous Mentoring

Topic	Key Learning Points
Academic Advancement	Develop a niche and become an expert in a small or unpopular area of medicine. Have a systematic approach with milestones for academic promotion. Start early in residency and work with faculty to start a project. Say yes to opportunities even if you're not an expert. Network with peers at other institutions, learn from obstacles and barriers, appreciate the journey. Academic advancement is important and can lead to recognition and certain leadership positions.
Mentorship Development	You need different mentors for different aspects of your career development. Find mentors who are willing to give their time and help you grow. Take advantage of speaking engagements to make contacts and build your network. Finding your niche involves identifying what information is missing in your field and coming up with a good research question to answer. Collaborate with colleagues and mentors to carry out research projects. Don't hesitate to switch mentors if they are not providing the necessary time and resources to help you.
Leadership Development	Find like-minded individuals with similar principles, ideas, and expertise to build a network inside and outside of your organization. Volunteer for leadership opportunities, such as being a member of a committee, to gain recognition and advancement in your field. Be cautious about taking on leadership roles too early in your career, as it can derail your academic productivity. Communicate with your team about the mission and goals to ensure their investment, determination, and engagement in the process.
Research Resources	Collaboration: Finding others to collaborate with and always having multiple projects in progress. Time management: Allocating time beyond patient care for research, writing, reflecting, and analyzing. Funding: Finding mentors to help with funding and being persistent in submitting grants. Protected time: Asking for protected time to do research and education activities, starting with small grants early in your career.

Videography

An experienced videography team from the media support department was engaged to produce high-quality videos. During the recorded filming sessions, each mentor was asked questions appropriate to their experience and specialty and provided answers from their perspective. The media support team provided editing and formatting, keeping each video under 3 minutes in length.

Video series distribution

The compiled video series comprised 24 modules, featuring 99 short videos. Each module focused on a specific question and incorporated perspectives from several mentors. The videos were shared in March 2020, through posting on our institution’s intranet site and emailing to several distribution lists within our organization, including the Department of Medicine. Feedback was sought from viewers of the video series. 

Data collected

Analytic reports were generated to track key metrics since the initial posting of the videos. These metrics generated included the count of unique viewers, the total number of videos viewed, and the total duration in minutes of viewership. 

A survey was sent to all viewers consisting of four questions regarding 1) overall quality of the videos, 2) potential to enhance academic productivity, 3) other suggested topics, and 4) general comments. Between December 2021 and September 2023, the survey was distributed to all viewers via email using the Research Electronic Data Capture (REDCap) survey platform. Respondents were not required to provide personal identifiers, such as race and gender. Qualitative and quantitative responses were gathered from the survey participants.

The project involves quality improvement and determined by the IRB reviewer that it does not constitute research as defined under 45 CFR 46.102. Continued IRB review of this project is not required.

## Results

Between March 2020 and September 2023, the VAAM Video Series garnered 182 unique viewers, received 2,107 visits, and accumulated 1,871 total minutes of viewing time. Of the 182 viewers, 21 completed the survey (11.5% response rate). Forty-three percent of survey respondents provided comments (n = 9/21).

Quantitative results

**Figure 1:**
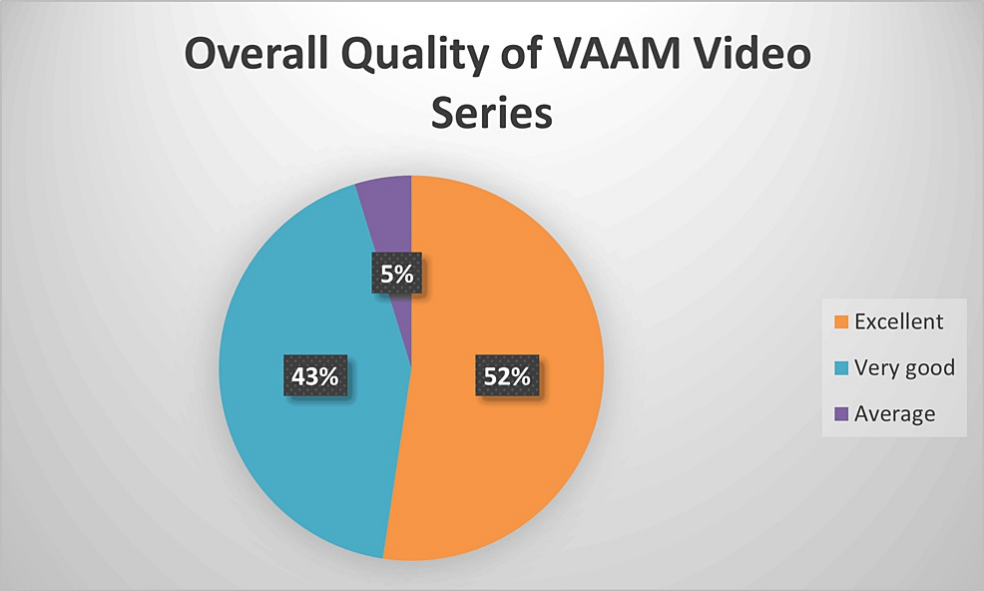
Survey respondents' perspective of the quality of the Virtual Academic Asynchronous Mentoring (VAAM) video series (content and practical applications)

**Figure 2:**
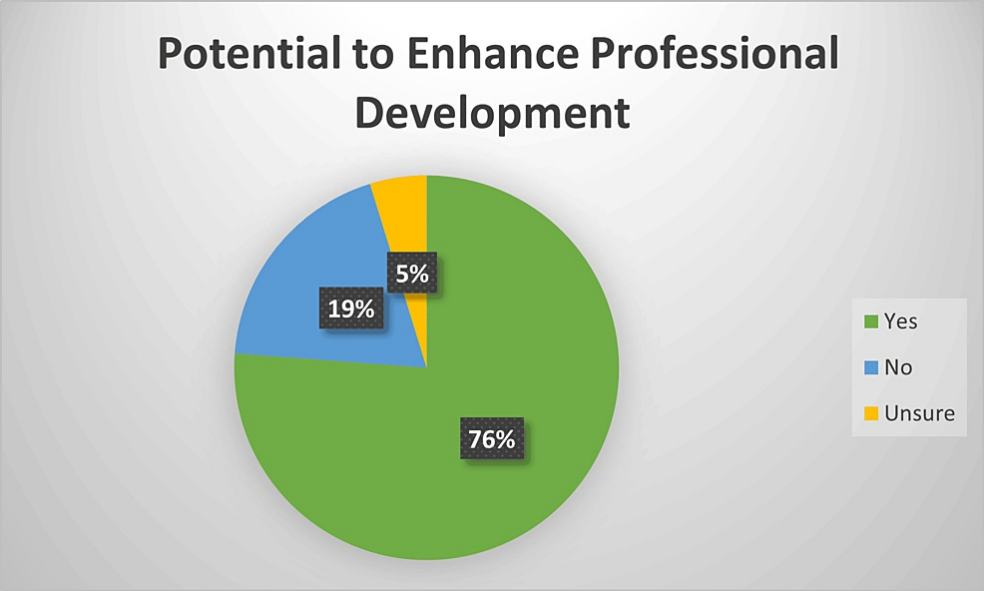
Survey respondents' perspective of whether the video series had the potential to enhance their professional development and academic productivity.

When asked about the overall quality of the VAAM video series including content and practical applications, 52% (n = 11/21) reported they were excellent, and 43% (n = 9/21) reported very good (Figure 1). Additionally, the vast majority of respondents thought the video series had the potential to enhance their professional development and academic productivity (76%, n= 16/21), while only 19% (n= 4/21) felt it did not (Figure 2).

Qualitative results

General comments provided by respondents noted the benefits of creating access for those without established mentorship, improving professional development, offering motivational advice, and convenience as well as future topics to consider (Table 2). 

**Table 2:** General comments shared by viewers

Themes	Comments
Creating access for those without established mentorship	“VAAM provides valuable support and guidance to mentees and new faculty members who lack established mentorship relationships.” “This is simply awesome, especially in smaller departments with not as many senior consultants that can mentor others.”
Improving professional development	“By offering access to academic promotion and mentorship guidance, this series effectively bridges the gap in mentoring and quips individuals with essential information.” “The video series has potential to extend beyond its current scope, as it can serve as a model for creating similar programs to reach faculty members in diverse locations, fostering their professional development across Mayo Clinic.”
Offering motivational advice	“I love those videos and the topics. It makes it so real and personable to hear the stories of highly accomplished PI in the virtual world. It helps the mentees feel motivated.”
Access and convenience	“Thanks to everyone who is part of this project. Having it all in one site and the user can select the leader or position they are interested is wonderful.” “I have personally shared this program with my mentees and have found that videos to be an invaluable time-saving tool.”
Future topic suggestions	Leadership, Teaming, and Communication How to achieve academic advancement with a focus on the medical education pathway to tenured position and understanding institutional criteria How to perform research without grant funding and identifying other sources of financial support How to identify successful research mentors with sufficient time and commitment to invest

## Discussion

The VAAM video series was developed and prepared prior to the beginning of the COVID-19 pandemic and was fortuitously released March 2020 when COVID-19 first began spreading widely in the United States. The virtual mentorship model enabled mentorship to continue despite the need for physical distancing during the pandemic. Additionally, with virtual learning becoming more mainstream during the pandemic, our target audience of faculty physicians was already becoming familiar and comfortable with virtual learning models. The virtual platform provides convenient accessibility to numerous faculty members while connecting individuals from diverse backgrounds and locations and is adaptable to multiple different practice settings and locations.

Although little has been written about virtual mentoring for faculty physicians’ academic development, our study suggests that virtual asynchronous mentoring using video interviews with experienced and successful faculty members could be a valuable tool for academic growth and guidance. We found that the vast majority of VAAM survey respondents were favorable that the content was practical and helpful (95%) and felt their professional development and academic productivity would benefit (76%). Due to our survey's anonymous nature, verification of race and gender information from our respondents was not possible. Consequently, the outcomes may not accurately represent the viewpoints of women and underrepresented faculty. It is also noted that our survey cohort numbers were too small to achieve statistical significance, though the survey was intended to be qualitative feedback for future improvements. There is no direct comparison of previous studies to the asynchronous aspect of our VAAM model, as prior available studies have been virtual but synchronous [21,23,24]. 

Reviewing the disparities data for academic promotion for women and underrepresented in medicine, there is significant potential benefit in expanding the VAAM approach as VAAM has the potential to lessen the gender and racial gap in academic medicine and academic promotions by ensuring all mentees have access to the same guidance and advice without unconscious bias or social barriers [9]. VAAM additionally allows an open forum for mentors to share a range of diverse perspectives and experiences giving mentees a broader scope of learning.

Notably, VAAM allows mentees to choose specific topics catered to their interests and needs, and the asynchronous nature also enables them to revisit and reference the content more than once. The top three conversations for VAAM participants centered on career development, research, and promotion. Although most faculty are interested in their career development and promotion, junior faculty are often particularly focused on early academic promotion, especially those in the time-limited tenure academic track [5]. While large academic medical centers may have numerous experienced faculty mentors available, this may not be the case at other institutions. Even within academic medical institutions, certain divisions or certain shifts may have less access to synchronous and scheduled mentorship opportunities.

Based on the definition of Merriam-Webster dictionary, mentorship is "the influence, guidance, or direction given by a mentor" [28]. It is recognized that the personal connection and real-time feedback that happens during traditional mentoring relationships offer unique and invaluable benefits that cannot be fully replaced by virtual mentorship, including the potential for coaching style questions from the mentor to the mentee and personalization or tailoring of advice to each unique individual’s situation. There are also limitations to the traditional dyadic mentoring relationships, and not all academic medical centers have created institution-wide mentoring programs [5]. VAAM provides an adjunct to traditional mentorship and has its own unique advantages that could be adapted at many different institutions cost-effectively.

## Conclusions

Virtual mentoring utilizing an asynchronous video platform offers a novel approach to academic mentoring and career development which bypasses some limitations of traditional mentorship models in a convenient and cost-effective way. Virtual mentoring may serve as a starting point for individuals who have not yet established a traditional mentoring relationship, as well as for those who already have a traditional mentor in place but wish to augment and expand their mentorship scope. VAAM is an excellent alternative to and augmentation for traditional mentorship by providing egalitarian access for mentees, and diversity of thought from mentors, in an asynchronous format, in an efficient and explorable manner.
